# Living and dying with Brenda Walker (and Motor Neurone Disease)

**DOI:** 10.1177/23982128251363409

**Published:** 2025-07-31

**Authors:** Brenda Walker, Claire Durrant, Kate Baker

**Affiliations:** 1 Tudor Lodge Residential Care Home, Somerset, UK; 2 Centre for Discovery Brain Sciences, The University of Edinburgh, Edinburgh, UK; 3 UK Dementia Research Institute, The University of Edinburgh, Edinburgh, UK; 4 MRC Cognition and Brain Sciences Unit, University of Cambridge, Cambridge, UK; 5 Department of Pathology, University of Cambridge, Cambridge, UK

## Letter – June 2025

**Figure:**
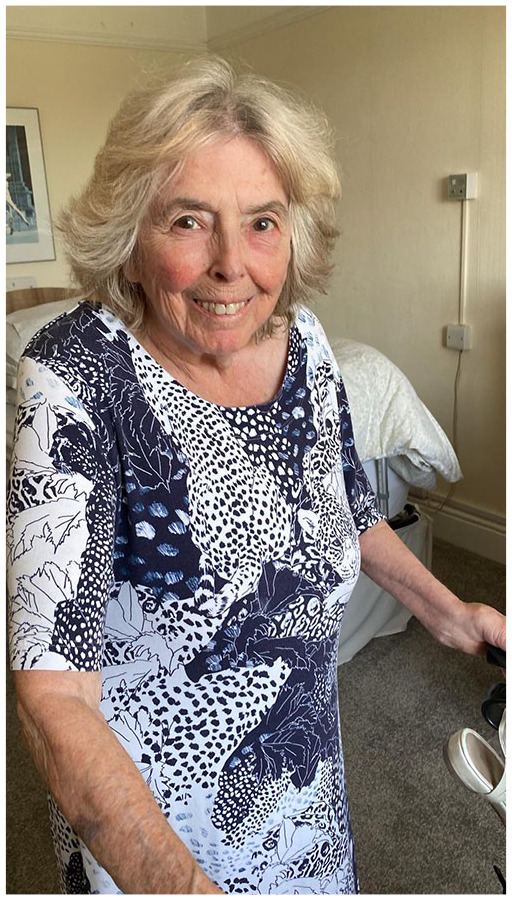


My name is Brenda Walker and I am now 91, but still a resident reviewer for the British Neuroscience Association. Now, ironically, I have been diagnosed with Motor Neurone Disease (MND), described by the neurologist as Anterior Horn Cell disease, an aspect of Amyotrophic Lateral Sclerosis. In this terminal disease, the damage to motor neurons disrupts the transmission of electrical signals from the brain and spinal cord to the muscles, leading to progressive muscle weakness and atrophy. This disruption causes the muscles to lose their ability to contract and respond to commands from the nervous system.

Apparently, it has been developing in me slowly since I had a small silent stroke in the cerebellum during May last year, not diagnosed until the November of 2024. This affected the fingers of my left hand after which I experienced ataxia that slowly crept up my left arm to the elbow. The physiotherapists from the Stroke Association discharged me at the end of January 2025 saying how well I had recovered. However, in February, everything seemed to go in reverse and the ataxia travelled up to the shoulder and neck. Having seen local doctors, it was not until, as a matter of urgency, I sought a neurologist privately, had a second magnetic resonance imaging (MRI) and finally a nerve conduction test, that I was given a definitive diagnosis of MND.

Already, the weakness has spread to my legs causing me to fall a week ago. Since then I can no longer walk unaided and need a micro-provider to come each day to wash and dress me. Already I can feel my neck and throat muscles tightening and an over production of saliva when I eat. Each day, I feel a little weaker.

I would like to take this opportunity to invite all those not in favour of assisted dying to imagine you are in my shoes. You will have read with horror some of the atrocities happening in torture chambers round the world where restraints prohibit movement; where the prisoner has no voice; where breathing in gasps and choking on food are normality. Prisoners locked in Hell. Anyone with MND has such a future vividly in mind.

‘Oh’ I hear you thinking, ‘But the hospices and carers are wonderful, loving and kind. The environment is pleasant and you will be well supported medically with palliative care on this final journey’.

But today I am taking YOU on a journey. Today ‘YOU’ are ‘ME’, and here is a 10 point breakdown, or rather a slow build-up, to your own death.

1. First one limb, in your case, your arm, becomes stiffened. You can no longer use it to even scratch your nose, eat a biscuit, cut up your food, drive, or play the piano.
2. Your legs begin to feel weak, you fall, then cannot walk unaided.
3. You notice your throat swelling, a painful neck tightening and a vast increase in saliva so you drool a little when eating.
4. You now need help to get in and out of bed. Remember, you can only use one arm, and most mornings you have severe cramp in at least one leg or foot. Your heels feel on fire and until you stand upright there is a sizzling sensation in your toes, and your balance is unsteady until you can hold onto something.
5. What now lies ahead? Your voice starts to change due to the tongue muscle being affected.
6. The intercostal muscles in your thorax weaken so you are struggling to breathe, choking on any food you try to eat (with your right hand that so far still works). Eventually you can no longer communicate with speech.
7. You notice a weakness in the fingers of your right hand.
8. It travels to your shoulder.
9. You are now incapable of doing anything yourself. You are LOCKED IN. Locked into the prison of your own body.
10. AND LASTLY, all toilet needs work internally, but you can do nothing apart from see and hear those kind carers, when you are just BEGGING to end the torture of this cruel terminal disease.

I am writing this not only for myself, but for all those suffering from MND or other terminal illnesses, those who are helpless, unable to beg for themselves. I write now, before I too can no longer communicate the fact that I have had a long, happy and successful life with a large loving family and would like to die with dignity, while capable of communicating the love I have for them all.

## Postscript – July 2025

So, the diagnosis has been made and the patient’s reaction described above. Eduardo Mercado III (2024) comments that as researchers find the ‘mind’ hard to define, they conclude it to be the ‘hidden parts of a mental activity’ (Chapter 1. ‘The Science of Minds’, p. 5). The patient’s conscious mind is thinking, perceiving, remembering, feeling, and willing the whole process of slow dismantlement to disappear. The patient’s unconscious mind however, is in a state of chaos.

Hengen and Shew (2025) suggest a new holistic approach to understanding the workings of the brain and mind. The brain is said to be at its most operative when at ‘near-tipping’ point. Criticality is a term taken from physics when describing this complex system, where even small changes can activate a massive chain reaction. This concept is found not only in physics, but also in neuroscience, engineering and social sciences. Hengen and Shew propose that criticality is a ‘setpoint’ where homeostatic mechanisms are brought back from chaos to perfect balance. The authors believe that one way to restore criticality may be sleep and, if so, this could be a new route to tackle neurodegeneration. In MND and Parkinson Disease, a high percentage of cell death affects dopaminergic neurons and synapses, occurring prior to any conscious symptoms. Thus ‘the disruption of function central to any neurological disease is defined by the point at which homeostatic mechanisms [such as dopaminergic transmission] are no longer capable of maintaining functionally relevant setpoints’.

*But the patient is not interested in computation*

*Her own chaos sheds tears at the destruction,*

*Unseen, stealthily ravaging dopamine receptors,*

*Synapses, the electrical song along motor neurons*

*Muted. Hidden beneath unknowing skin, waiting*

*For the tragedy to begin.*

*And now the chaos of a home abandoned*

*A love, abandoned. What to take? What to wear?*

*What to leave when I go into Care? How long*

*Before I no longer control my tongue*

*And words remain imprisoned*

*In my head. For how long?*

*Maybe, just maybe, in writing this,*

*The balance of my brain has shifted.*

*Objects are carefully placed,*

*A new day has dawned.*

*Beyond my window another garden,*

*The see-saw of my brain, settled,*

*Stabilised. I am creative again.*

*I am learning again.*

*There is order.*

## Editorial comment

### What are the neuroscience research challenges arising from Assisted Dying?

The Terminally Ill Adults (End of Life) Bill (UK Parliament, 2025) is a proposed law in England and Wales that will ‘allow adults who are terminally ill, subject to safeguards and protections, to request and be provided with assistance to end their own life’. Inspired by Brenda’s experience and writing, we wish to highlight the following high priority clinical research challenges posed by the Assisted Dying Bill:

- Assisted Dying will become an available choice for an individual with an inevitably progressive, irreversible illness when death can reasonably be expected within six months.
  - How accurately can a six month survival time (prognosis) be predicted for the diverse range of terminal neurological and neurodegenerative conditions?
  - Do we have sufficient understanding of the factors influencing each individual’s illness trajectory, to know what information is required for reliable predictions?
  - When a condition is rare and data are limited, or prognosis is less predictable, will the Assisted Dying choice be unavailable?
  - How might existing treatments, whether disease-slowing or palliative, intersect with prognostic accuracy and the availability of Assisted Dying?

- Assisted Dying will require self-administration of life-ending medication. People with advanced neurological conditions, like MND, are significantly disabled and may be physically incapable of self-administering life-ending medication.
  - Will individuals who are physically incapable of self-administration be discriminated against in accessing this choice?
  - Could any alternative Assisted Dying mechanisms be made available to them?

- An individual can only be considered eligible for Assisted Dying if, at the time of the request, they have capacity to make this decision.
  - What if cognition is significantly, irreversibly impaired by their terminal condition, such that they no longer have capacity to make this choice or can no longer effectively communicate their view, but are very significantly distressed by the prospect of an end-of-life without autonomy?
  - In the context of a progressive cognitively-impairing neurological condition, when and how can capacity be established for this particular choice?

- There is understandable concern that a treatable psychiatric illness could cloud an individual’s judgement or temporarily influence their wishes about Assisted Dying. However, differentiating between symptoms of a psychiatric illness and symptoms of a neurodegenerative condition may, sometimes, be an illogical as well as an impractical dichotomy–the untreatable terminal illness may itself directly (via brain dysfunction) or indirectly (via psychological response) cause the psychiatric symptoms.
  - Might denying or delaying the option of Assisted Dying because of the presence of mental distress, despite meeting other criteria, magnify and prolong suffering?
  - What conceptual and methodological strategies can be developed to (a) navigate the judgement of capacity and (b) meet the complex care needs of individuals with neurodegenerative conditions and co-occurring psychiatric symptoms?

- In parallel with the proposed changes to end-of-life options, treatment trials are underway for some neurodegenerative conditions, which may slow down the course of a condition or halt its progression, while not reversing damage done.
  - Might patients have to choose between embarking on an experimental treatment or leaving-open the possibility of Assisted Dying?
  - And, if so, might this impact on clinical trial recruitment and discovery of treatments that may benefit patients at an earlier stage of the condition?

We call on members of the British Neuroscience Association, and the wider neuroscience research and clinical communities, to engage with these challenging questions. Please identify and address additional questions we have not even considered.

Solutions are needed, urgently, to improve end-of-life care, including fair access to Assisted Dying, for patients with neurological conditions.

## Leaving the last words to Brenda

*Words, words. . .*

*The patient’s been listening;*

*No-one’s mentioned timing,*

*The waiting for appointments.*

*It’s not the end we worry about*

*It’s the route, the way,*

*Grasping at straws as the day*

*Draws to a close.*

*Then the news*

*Four new trials to begin,*

*They think they’ve found a way*

*To cure MND*

*Four trials to start -*

*In London though.*

*So far, so far from me*

*Where the sun drags its feet,*

*To reach a cure for MND.*
